# Moldable Mask: A Reusable, Hot Water Moldable, Additively Manufactured Mask to Be Used as an N95 Alternative

**DOI:** 10.3390/ma14227082

**Published:** 2021-11-22

**Authors:** Erica Martelly, Charles Li, Kenji Shimada

**Affiliations:** Department of Mechanical Engineering, Carnegie Mellon University, Pittsburgh, PA 15213, USA; cli3@andrew.cmu.edu (C.L.); shimada@cmu.edu (K.S.)

**Keywords:** mask, personal protective equipment, N95, 3D printing, customization

## Abstract

There has been high demand for personal protective equipment (PPE) during the COVID-19 pandemic, especially N95 respirators. Unfortunately, at the early stage of the pandemic, the supply could not meet the demand for N95 respirators, leading to a shortage and unsafe reuse of this form of PPE. We developed the Moldable Mask to ease the demand for N95 respirators by creating a 3D-printed mask that uses a piece of N95 material as a filter. A sheet of N95 material could be used or one N95 respirator to be turned into two masks. The main feature of the mask is the ability to easily mold it in hot water to create a custom fit for each user. It can also be easily assembled at home with affordable materials. The final mask design was qualitatively fit tested on 13 subjects, with all subjects showing an improvement in fit with the hot water molding technique and 10 (77%) subjects passing the fit test. This shows that the Moldable Mask is a viable option for a safe, affordable N95 alternative when N95 mask supply is strained.

## 1. Introduction

Throughout the COVID-19 pandemic, there has been high demand for personal protective equipment (PPE) in the United States, notably N95 respirators [1,2]. These respirators are a critical piece of PPE considered to be critical in preventing inter-human infection from the virus [3,4]. Even in November 2020, ten months into the pandemic, 61% of facilities reported shortages of PPE [5]. Furthermore, due to the ongoing shortage, many healthcare workers resorted to reusing N95 respirators, despite them being designed as single-use items [4,6,7,8]. Efforts have been made to ease demand by extending the life of N95 respirators using sterilization [4,7,8,9,10], but there is evidence that certain sterilization techniques can degrade respirator efficacy [8,10] and continued re-use can lead to respirator fit failure, putting healthcare workers at risk [11,12]. Despite efforts by companies, the U.S. government, and nonprofit organizations, the supply has often not been able to reach the demand. The increased usage of disposable PPE has not only stressed the supply chain but has also led to a large increase in medical waste. For example, hospitals in Wuhan produced six times more single-use plastic-based medical waste than before the pandemic [13]. This increased waste has the potential to negatively affect the environment, especially marine life [14,15,16].

An additional challenge posed by N95 respirators is that they must be fit properly to ensure respiratory protection. Improperly fitting respirators reduce the amount of respiratory protection provided by the device which could lead to infection [17]. Not all respirators fit all faces, and users will typically try different respirators and sizes to find a good fit [18,19].

One way to address the issue of respirator shortage and PPE waste is to use additive manufacturing (AM) to create 3D printable reusable respirators with replaceable filters. N95 materials would still be necessary for the filter, but one N95 respirator could be used for multiple 3D-printed ones or a roll of N95 material could be used instead. This reduces strain on the respirator supply and decreases consumption. Various groups and individuals have worked on developing 3D-printed PPE [20]. One of the most successful is the Montana mask which is now being injection molded. Although this mask has passed an OSHA fit test on one person, there is not much information on whether this mask can pass fit testing for a greater number of individuals [21]. Another group is the Barrow Neurological Institute that has developed a method that uses 3D printing and silicone casting to make a customized mask. This allows for a much better fit but is a time-consuming process. Furthermore, the fit cannot be adjusted after the mask is completed, which could lead to a lengthy re-fitting process if the mask does not fit well [22].

There are also other 3D printable masks described in the literature to address the N95 mask shortage. For example, one group from Stanford developed a silicone 3D-printed mask that uses a quarter section of a rigid N95 mask as the filter [23]. They quantitatively tested the mask on six subjects, who all passed the fit test. However, their mask design uses 3D-printed silicone, which requires an expensive, specialized 3D printer, making it difficult for a home maker to print and use. Another group at the University of Kansas created the Kansas City mask, which, like the Moldable Mask, offers the use of thermoforming to improve the mask fit and uses liquid rubberized sealant to add cushion to the mask edge [24]. The authors state that their mask passed a qualitative fit test which relies on taste and smell to determine mask fit but do not state how many subjects they used.

The 3D printable mask presented here, named the Moldable Mask, seeks to address the issues that these other 3D printable masks have not yet tackled. The mask is easy to custom-fit just using hot water and features a large filter space to make it easier for the wearer to breathe while using the device. It is easy to print on any fused deposition modeling (FDM) printer and assemble with affordable supplies such as weather seal. It has also been quantitatively fit tested on 13 subjects. All subjects had an improved fit with the hot water molded masks and 10 subjects passed the fit test.

## 2. Materials and Methods

### 2.1. Initial Mask Design

**Figure 1:**
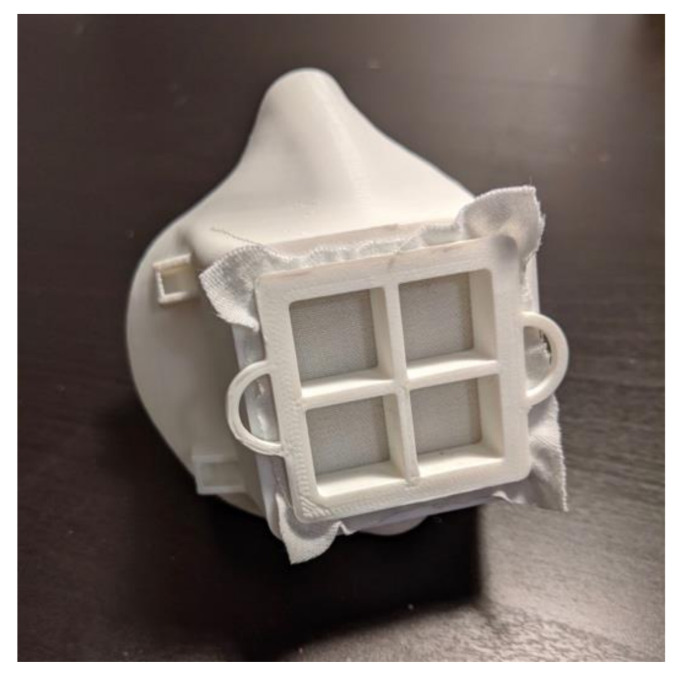
First iteration of the Moldable Mask.

The mask presented here was initially designed as part of the Fit to Face Mask Design Challenge by America Makes in May 2020. The goal was to create a face mask design that could fit a wide range of faces and seal well onto the users face while being optimized for 3D printing [25]. The first iteration of the mask was chosen as one of the top designs in the challenge [26]. The 3D model files and instructions for use have been available since June 2020 on the NIH 3D print exchange website [27] and on a Carnegie Mellon University webpage [28]. The first iteration is shown in Figure 1.

The key feature of this mask is its moldability. The idea of a moldable design was inspired by our previous work in designing Custom-Fit CPAP masks to provide an improved fit and seal [29]. We decided to create a moldable mask to implement custom fitting in a rapid and easy way. In order for the mask to be moldable, it must be produced in a material with a low glass transition temperature and low heat deflection temperature. For this reason, the mask is printed in polylactic acid (PLA) filament, which has a glass transition temperature of 61 °C [30] and a heat deflection temperature (HDT) of 49–56 °C [31,32]. Before designing the shape of the mask, three strips of 3D-printed PLA plastic were 3D printed at three different thicknesses: 1.5 mm, 2 mm, and 2.5 mm. The pieces were submerged in hot water for 15 s and then deformed manually. The 1.5 mm thickness was chosen since it was the easiest to deform but still maintained qualitative structural integrity. Now knowing the desired thickness of the mask, the first iteration of the mask was designed in SolidWorks CAD software (Dassault Systemes, Version 2020, Vélizy-Villacoublay, France). The shape of the contour that touches the face was loosely based on the Montana Mask with some modifications, mostly in the nose area. The nose point is shorter and the curve at the nose is also different as a result. This face-interacting contour is then lofted to the square filter space. The initial filter space was a 40 mm square. The first iteration of the mask was also designed to print without support material.

**Figure 2:**
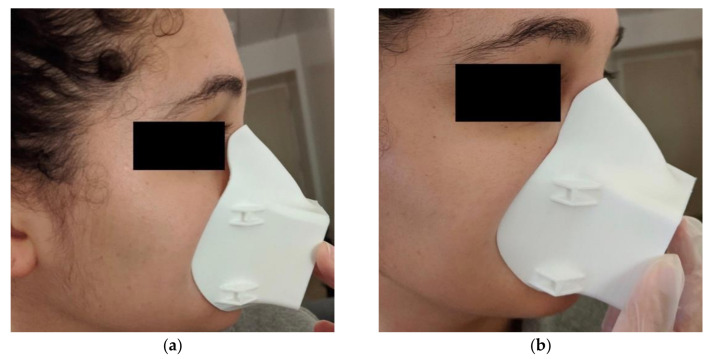
First iteration of the Moldable Mask: (**a**) before hot water molding and (**b**) after hot water molding.

The first mask iteration was 3D printed in 1.75 mm PLA filament on a Monoprice MP Select Mini 3D Printer with 25% infill and 0.175 mm layer thickness. This first mask was tested by one of the authors in their home to determine the viability of the hot water method. To mold the mask, we start by preparing hot water to about 70 °C to reach the glass transition temperature of PLA. The face edge of the mask is dipped in the hot water for 10–15 s to soften. The mask is then immediately placed on the user’s face where they use their fingers to adjust the fit, taking care around the nose area in particular. The process can be repeated as needed. As shown in Figure 2, this method allows adjustment of the mask contour to better fit the person’s face.

### 2.2. Final Mask Design

**Figure 3:**
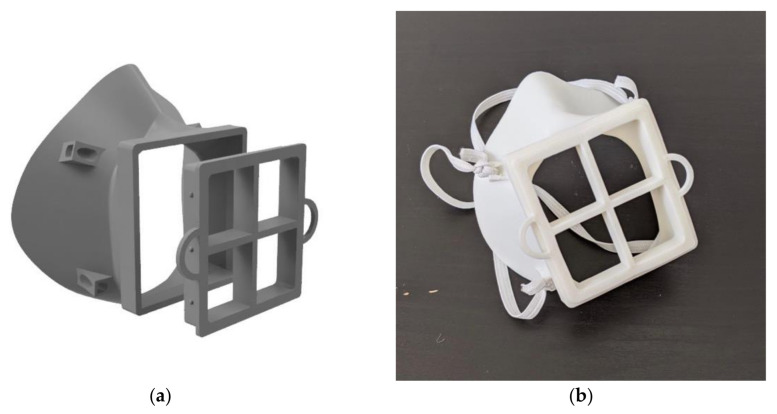
Final version of the moldable mask: (**a**) 3D rendering and (**b**) physical version.

**Figure 4:**
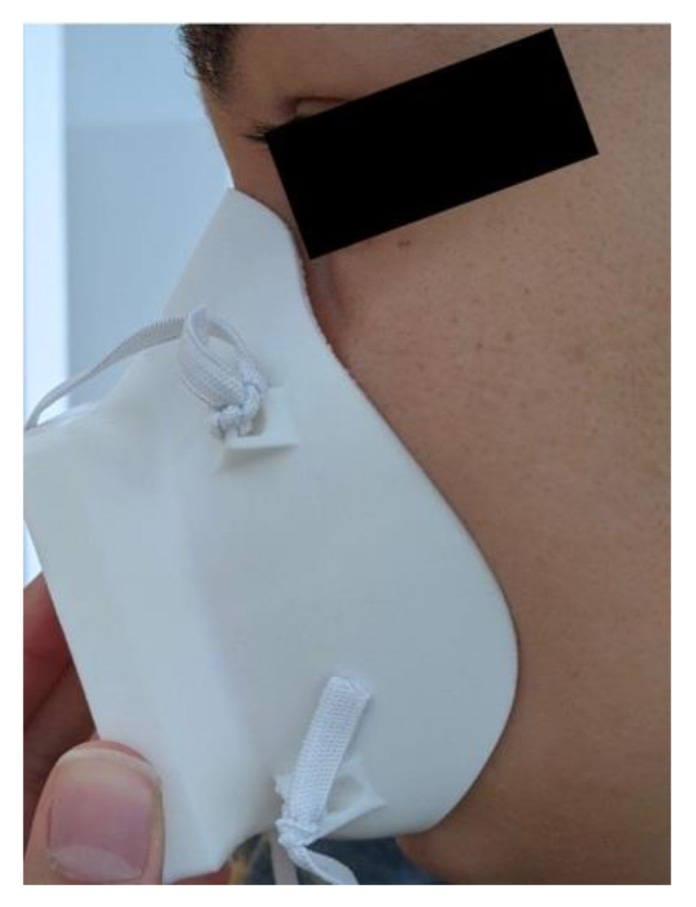
Close up of unmolded nose contour on the final mask version.

An initial fit test of this first mask iteration revealed areas for improvement which were addressed in the subsequent iteration. Three key changes were considered: increased filter size for improved breathability, adjustment of the nose contour and increased mask body size. When testing the first iteration, it was difficult for the user to breathe, which would not only make daily activity more difficult but also affect the seal of the mask against the face. Difficulty breathing led to more forceful exhales, which pushed the mask away from the face and caused leakage around the sides. In terms of the nose contour, as can be seen in Figure 2a, the nose contour is initially quite far from the face. This contour was adjusted to sit closer to the face, therefore making it easier to mold the mask. The mask was also slightly too small and sat too high on the user’s chin, making it difficult to speak and move their mouth. The medium mask size, on which all the other designs are based, was scaled up by 5% to fit more comfortably. Several iterations of the mask were created taking these changes into account and qualitatively tested, until the final mask design was selected, shown in Figure 3. All designs after the first iteration were modeled in Autodesk Fusion 360 (Autodesk Inc., Mill Valley, CA, USA). The final mask has a 57.5 mm × 62.5 mm filter size, which is an increase in area from the first design of 225%. This was the largest filter size we could manage without compromising the body of the mask. This makes breathing with the mask much easier. This change in the breathing area resulted in requiring support material for the 3D printing process. The nose contour was also adjusted, as shown in Figure 4. The orientation of the strap loops was also modified to have the top strap sit higher on the head. This mask was printed on a Raise3D Pro Plus (Raise3D, Irvine, CA, USA) printer in 1.75 mm TRUE Food Safe PLA filament (Filaments.ca, Mississauga, ON, Canada) with 40% infill and 0.2 mm layer thickness. One concern with any manufacturing process is part accuracy. We did not experience any obvious defects or failures when printing masks for testing. Furthermore, this mask is intended to be deformed, so part accuracy is not critical to having a well-fitting mask.

Multiple sizes of the mask were also created to accommodate varying face sizes and features and make the mask easier to mold for varying facial features. The mask sizes are small, medium, large, medium-narrow, medium-wide, and medium-flat. The medium-flat mask has a flatter nose area to make the mask easier to mold for people with lower nose bridges. All medium masks have a height of 101.2 mm. The widths are: medium—98.8 mm, medium-narrow—92.7 mm, medium-wide—104.3 mm, medium-flat –98.8 mm. The small mask has a height of 92.5 mm and a width of 88.8 mm. The large mask has a height of 112.8 mm and a width of 109.0 mm. The six mask sizes all use approximately the same amount of material, on average 39.0 ± 2.5 g of PLA including support material. More specifically: small—38.0 g, medium—37.9 g, large—44.0 g, medium-narrow 37.3 g, medium-wide 39.2 g, and medium-flat—37.8 g. The filter insert requires 15.3 g of PLA.

### 2.3. Mask Assembly

**Figure 5:**
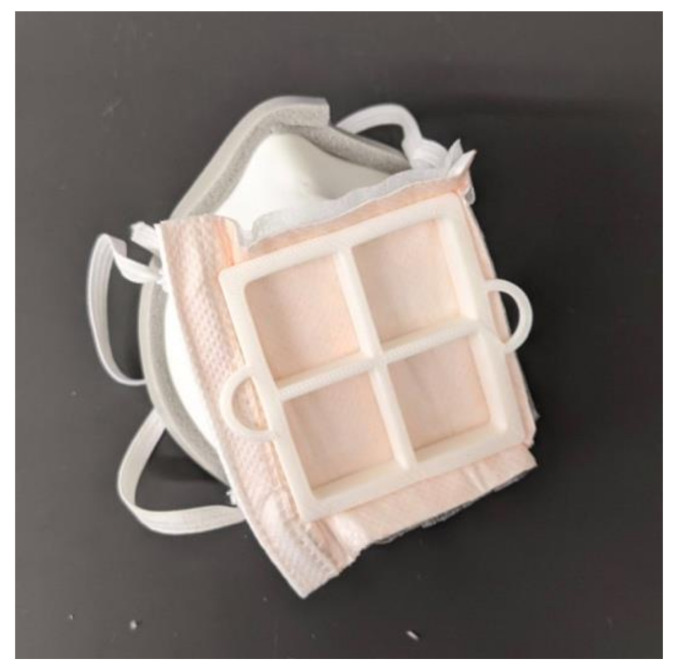
Fully assembled final version of the mask.

**Figure 6:**
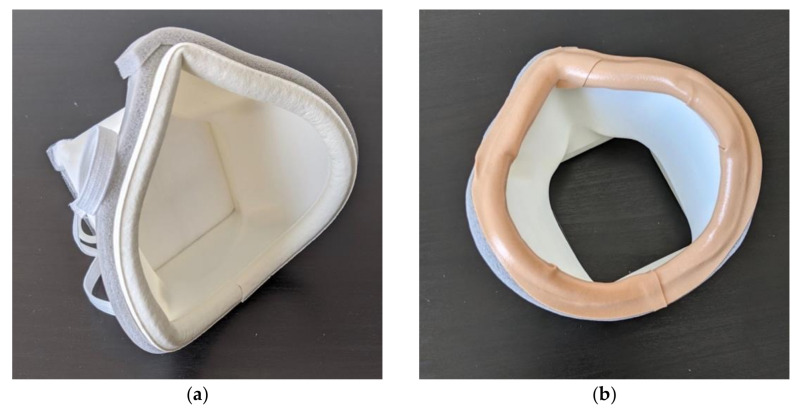
Sealing around the edge of the mask: (**a**) showing the weather seal material and (**b**) completed seal with skin-safe waterproof tape.

For these masks to provide protection from the COVID-19 virus, they must be used with N95 filter material. We used Kimberly-Clark N95 Particulate Filter Respirator and Surgical Mask Regular size (Kimberly-Clark, Irving, TX, USA) donated by Allegheny General Hospital. The user can use the filter insert as a guide to cut out an appropriate amount of filter material. This material is then press-fit into place on the mask, shown in in Figure 5. Cushioning material is added around the face-contacting edge of the mask to make the mask more comfortable and create a better seal. Additional sealing is added around the edge of the mask in case there are any gaps in the adhesive. The sealing is shown in Figure 6. A variety of materials could be used as cushioning, but we used Frost King 5/16-inch D-section silicone weather seal (Thermwell Products Co Inc., Mahwah, NJ, USA) on the interior of the mask and Frost King 3/8-inch vinyl foam weather seal to seal the gaps around the outer edge of the mask. A layer of Nexcare Absolute Waterproof Tape (3M, St. Paul, MN, USA) is then added to the edge of the mask to ensure that all materials contacting the face are skin safe. A medium size mask requires approximately 31 cm of silicone weather seal, 36 cm of vinyl foam weather seal and 33 cm of waterproof tape. To adjust the straps the user ties the straps to one side of the mask and then puts the mask on. Then the straps are adjusted to a snug fit and knotted in place. Once the straps are adjusted, the mask is complete. The fully assembled mask is shown in Figure 5. During regular use, the filter material, cushioning, and straps can be replaced daily. The plastic body and filter insert can then be prepared for reuse by sanitizing. The layered nature of 3D-printed parts using fused deposition modeling (FDM) as well as the low glass transition temperature of PLA can make these parts challenging to sanitize. Other researchers have investigated ways to sterilize 3D-printed PLA parts. Armijo et al. suggest briefly placing the parts in a dilute bleach solution and leaving to air dry, a method that proved to successfully decontaminate 3D-printed PLA parts from E. coli and S. aureus [33]. Other techniques that can be used on 3D-printed PLA that are available in hospitals include Ethylene Oxide sterilization [34], gamma radiation and electron beam radiation [35]. It is unclear how many times these sterilizations could be repeated before the mask begins to degrade, so great care should be taken to stop use of the mask once it shows signs of wear or degradation.

### 2.4. Mask Fit Testing Procedure

To function as PPE for medical workers, the masks need to pass a fit test that indicates that the mask seals well on to the user’s face to prevent particles from entering or exiting through any gaps. One of the accepted standard OSHA protocols for fit testing is an ambient aerosol condensation nuclei counter (CNC) quantitative fit test [18]. This test compares the concentration of particles inside the mask to those outside of the mask. We used a quantitative fit tester (Portacount Respirator Fit Tester, TSI Inc., Shoreview, MN, USA) device with the OSHA Fast Filtering Face Protocol to conduct this test. The mask filter is punctured so that a probe can be attached to measure particulates in the mask while another probe measures the ambient air. A particle generator in the room supplements the ambient particle count with a nontoxic salt (NaCl) aerosol. Users are asked to perform four tasks while wearing their respirator or mask: bending over, talking, head side-to-side and head up-and-down. Once the test has started, the fit test takes 2 min 20 s to complete [18]. The device calculates a fit factor, which is the ratio of the average ambient particulate concentration to the concentration measured inside the respirator for each test (*ff_n_*). The maximum score for each test is 200. The overall fit factor is calculated using Equation (1) [18]:

(1) $$\mathrm{Overall}\;\mathrm{Fit}\;\mathrm{Factor}=\frac{4}{\frac{1}{ff_{1}}+\frac{1}{ff_{2}}+\frac{1}{ff_{3}}+\frac{1}{ff_{4}}}$$

For a respirator to pass the fit test for an N95 mask, the overall fit factor must be 100 or greater, with a maximum score of 200. For example, subject 4 had the following fit factors for their test: bending over (*ff*_1_) = 145, talking (*ff_s_*) = 93, head side to side (*ff*_3_) = 65, head up and down (*ff*_4_) = 171. Therefore, the overall fit factor for subject 4 is:

(2) $$\mathrm{Overall}\;\mathrm{Fit}\;\mathrm{Factor}\;\mathrm{Subject}\;4=\frac{4}{\frac{1}{145}+\frac{1}{93}+\frac{1}{65}+\frac{1}{171}}=103$$

## 3. Results

The Moldable Mask was tested on 13 subjects (7 female, 6 male, age 18–30, median 21) after consenting in accordance with Carnegie Mellon University’s Institutional Review Board (STUDY2021_00000047). The subjects chosen were a convenience sample to test the feasibility of the Moldable Mask. The subject demographics were 7 Asian, 3 Caucasian, 1 Black and 2 mixed race. Subjects were required to be 18 or older, without facial hair, and able to safely conduct the movements required in the fit test: bending down, talking, head side to side and head up and down. Each subject was assigned a number to protect their identity. The hot water molding procedure was changed slightly for subject testing. Subjects used a cotton cloth face covering when molding the mask on their face to protect their skin. The mask edge was also measured with an infrared thermometer to be 50 °C before being placed on the subject’s face to minimize the risk of skin discomfort. From our observations, 50 °C is the lowest temperature where the mask will still deform. No subjects reported any discomfort on their skin when molding the mask with this method.

**Figure 7:**
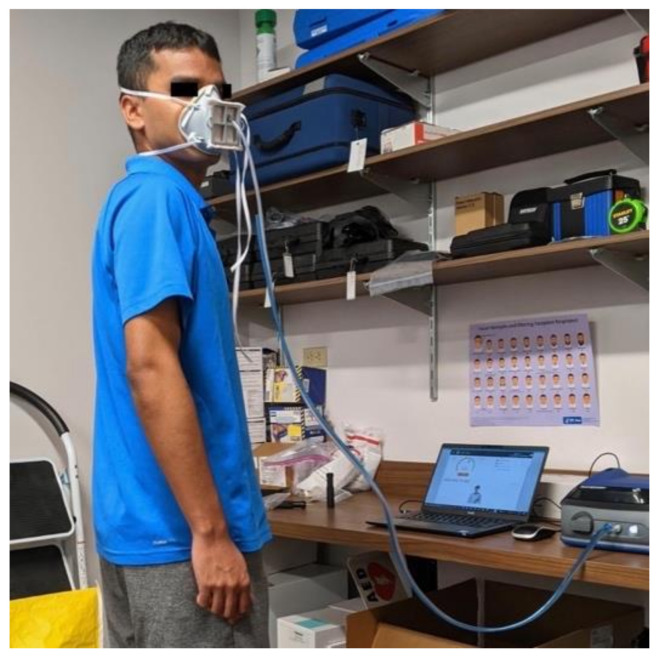
Subject wearing the Moldable Mask during fit testing.

Each subject tested the unmolded version of the mask and the molded version of the mask. The test could be repeated up to 3 times per mask and the best result was recorded. An example of a subject conducting the fit test is shown in Figure 7. All but three subjects use the medium size mask. One subject used the medium-narrow mask, one used the medium-wide mask, and one used the medium-flat mask. The overall results of the fit tests are shown in Table 1. The complete fit testing results are in Table S1. All subjects showed an improvement in fit between the unmolded and molded mask. The average unmolded fit test score was 7 ± 17 and the average molded fit test score was 143 ± 62. The average score for the molded mask in each test, bending over, talking, head side to side, head up and down were 166 ± 43, 134 ± 60, 142 ± 68, and 152 ± 70, respectively. The molded mask resulted in a passing score on the fit test for 10 out of 13 subjects (77%).

## 4. Discussion

We have described the development of an easily customizable, 3D-printed mask to function as an N95 respirator replacement. The mask can be made on any FDM printer with PLA filament, a cost-effective and accessible material. It is easily moldable at home with hot water to provide a good fit and can be assembled with affordable, everyday items. When tested with an OSHA-approved quantitative fit test most subjects obtained a passing score, which shows that the mask can provide respiratory protection. Furthermore, all subjects had an improved fit with the molded mask vs the unmolded one, showing that the hot water molding technique does improve the mask fit. PLA is the only commonly available 3D printable material with a glass transition temperature low enough to make it easy to mold at home without reaching risky temperatures. Polyethylene terephthalate glycol (PETG) is another material with a lower glass transition temperature and heat deflection temperature. However, with an HDT of 68 °C [36], the temperature needed to mold the mask is much higher than PLA at 51 °C. Users would need to protect their skin from these temperatures when molding if PETG is used.

There were three subjects that did not pass the fit test, although visual inspection showed a good fit of the mask on their faces. There are many possible reasons for this, but one key element to creating a successful fit is the strap tension and orientation. The mask must be worn very tightly, just before the point of becoming uncomfortable. It also helps to pull the top strap up to the crown of the head to pull the nose of the mask into the face and create a good seal. The subjects that had a failed fit test had short, smooth hair, which made it difficult to keep the top strap from sliding to the back of their head. This may have caused the seal to weaken and result in leakage during the fit test. Other individuals with long hair or short, textured hair avoided this by either using a ponytail or friction to keep the strap from sliding. One way to prevent this in the future is to use a different, rubbery, strap material that could stay in place more effectively due to its stickiness. The mask design could also be updated to change the location and orientation of the strap loops to allow for a better seal around the nose. This change in strap could improve the fit to a passing level, but additional testing would have to be done to ensure this is the case. This observation also highlights the importance of fit testing. Even though a mask may appear to have a good fit, the fit test can reveal leakage that would mean the user is not getting N95 level protection.

## 5. Conclusions

By leveraging additive manufacturing and the low glass transition temperature of PLA, we were able to create a 3D-printable Moldable Mask that, when combined with appropriate filtering material, can function as an N95 respirator. This mask can help address several issues such as mask fit, PPE shortages and medical waste. The moldability of the mask allows for customization of the mask contour so that it can fit a wide range of faces. The filter size allows the user to get two filters out of one N95 respirator or use sheets or rolls of respirator material instead. Finally, the reusability of the mask body helps reduce the amount of PPE waste that is produced and eases the demand for N95 respirators. As shown in testing, this mask can pass OSHA standards for N95 respirators for 77% of users tested, so wearers can be confident that they are being protected from harmful aerosols and particulates.

It is important to note that these masks have only been tested on 13 subjects so it is possible that the mask designs may not accommodate all face shapes and features. Furthermore, the masks were only used for about 5–10 min while preparing for testing and undergoing the fit tests, so they have not been evaluated for use throughout a full workday, which would be their expected use case. It would be valuable to have nurses and doctors tests these masks to see how they perform. Despite this, the Moldable Mask is an affordable, accessible way to provide custom-fit, reusable masks with N95 protection.

## Figures and Tables

**Table 1:** Overall fit testing results.

Subject	Sex	Molded Overall Fit	Unmolded Overall Fit
1	F	180	5
2	F	189	2
3	M	67	3
4	F	103	6
5	F	200	1
6	M	137	3
7	F	197	1
8	M	70	1
9	M	21	1
10	M	193	1
11	F	200	1
12	F	114	64
13	M	184	2

## Data Availability

Data are contained within the article or supplementary material. The data presented in this study are available in Table S1 at https://www.mdpi.com/article/10.3390/ma14227082/s1.

## Supplementary Materials

The following are available online at https://www.mdpi.com/article/10.3390/ma14227082/s1, Table S1: Complete fit testing results for all subjects.
