# Supplementary material for: Moldable Mask: A Reusable, Hot Water Moldable, Additively Manufactured Mask to Be Used as an N95 Alternative
Source: Materials (Basel). 2021 Nov 22;14(22):7082. doi: 10.3390/ma14227082 (PMC8624460; doi:10.3390/ma14227082)
Supplement: Supplementary file 1 [file materials-14-07082-s001.zip › materials-1425032-supplementary.pdf]

materials

Article

Moldable Mask: A Reusable, Hot Water Moldable, Additively Manufactured Mask to Be Used as an N95 Alternative

Erica Martelly *, Charles Li and Kenji Shimada

Department of Mechanical Engineering, Carnegie Mellon University, Pittsburgh, PA 15213, USA; cli3@andrew.cmu.edu (C.L.); shimada@cmu.edu (K.S.)
* Correspondence: emartell@andrew.cmu.edu

Citation: Martelly, E.; Li, C.; Shimada, K. Moldable Mask: A Reusable, Hot Water Moldable, Additively Manufactured Mask to Be Used as an N95 Alternative. *Materials* **2021**, *14*, x. https://doi.org/10.3390/xxxxx

Academic Editor: Christopher C. Berndt

Received: 30 September 2021
Accepted: 19 November 2021
Published: date

Publisher's Note: MDPI stays neutral with regard to jurisdictional claims in published maps and institutional affiliations.

Copyright: © 2021 by the authors. Submitted for possible open access publication under the terms and conditions of the Creative Commons Attribution (CC BY) license (http://creativecommons.org/licenses/by/4.0/).

Table S1. Complete fit testing results for all subjects.

				Molded Mask						Unmolded Mask					
Subject #	Sex	Age	Mask Type	Bending Over	Talking	Side to Side	Up and Down	Overall	Pass?	Bending Over	Talking	Side to Side	Up and Down	Overall	Pass?
1	F	30	medium	171	200	161	195	180	Y	8	5	4	4	5	N
2	F	24	medium	200	160	200	200	189	Y	2	3	2	2	2	N
3	M	21	medium	123	60	74	46	67	N	2	3	3	2	3	N
4	F	20	medium	145	93	65	171	103	Y	6	7	7	6	6	N
5	F	21	flatter nose	200	200	200	200	200	Y	1	1	1	1	1	N
6	M	19	medium	129	108	141	200	137	Y	5	3	3	3	3	N
7	F	24	medium	200	187	200	200	197	Y	1	1	2	3	1	N
8	M	24	medium-narrow	86	71	54	79	70	N	1	1	1	1	1	N
9	M	24	medium-wide	104	24	12	18	21	N	1	1	1	1	1	N
10	M	24	medium	200	174	200	200	193	Y	1	2	1	1	1	N
11	F	21	medium	200	200	200	200	200	Y	1	1	1	1	1	N
12	F	18	medium	200	118	129	71	114	Y	146	110	57	35	64	N
13	M	20	medium	200	148	200	200	184	Y	4	3	1	2	2	N
				Total Pass					10			Total Pass			0
